# Supplementary material for: A Likelihood Approach for Real-Time Calibration of Stochastic Compartmental Epidemic Models
Source: PLoS Comput Biol. 2017 Jan 17;13(1):e1005257. doi: 10.1371/journal.pcbi.1005257 (PMC5240920; doi:10.1371/journal.pcbi.1005257)
Supplement: S1 File — (TAR.GZ) [file pcbi.1005257.s014.tar.gz › HSPH_Online-SI-Revision/IPoi09/n100-mild/n100-mild_BENCH_summary.pdf]

peak -8	R0	$1/\Theta_2$	inf	inflog	newly	newlylog	Reff	AttackRate
mean	27.3	49.9	295.4	0.7	506.4	0.8	29.6	46.5
median	10.2	35.6	247.	0.6	326.2	0.7	13.6	33.1
Quantile .1	3.	6.8	133.4	0.4	164.9	0.5	3.5	10.6
Quantile .9	72.6	131.5	495.2	1.1	850.1	1.5	73.5	101.9
4	11	15						
length	50							

peak -4	R0	$1/\Theta_2$	inf	inflog	newly	newlylog	Reff	AttackRate
mean	15.5	36.8	270.3	0.5	173.9	0.4	14.7	35.2
median	12.2	21.	245.2	0.5	160.1	0.4	13.	29.1
Quantile .1	5.2	2.8	119.6	0.4	87.5	0.3	7.	11.3
Quantile .9	29.7	84.2	402.4	0.7	278.6	0.6	19.6	64.8
0	0	0						
length	50							

peak	R0	$1/\Theta_2$	inf	inflog	newly	newlylog	Reff	AttackRate
mean	66.9	192.6	413.8	0.7	89.	0.3	33.2	94.3
median	58.1	155.3	401.7	0.7	87.2	0.3	30.5	93.9
Quantile .1	40.6	61.9	209.1	0.5	56.	0.2	21.9	69.4
Quantile .9	107.1	335.5	565.7	0.8	119.9	0.3	46.2	110.
0	0	0						
length	50							

peak +4	R0	$1/\Theta_2$	inf	inflog	newly	newlylog	Reff	AttackRate
mean	62.	170.2	676.5	0.7	188.5	0.3	41.9	94.
median	61.5	156.7	526.1	0.7	145.6	0.3	41.3	91.9
Quantile .1	49.	112.2	260.9	0.5	80.1	0.2	34.4	77.6
Quantile .9	75.6	231.4	876.	0.9	230.6	0.5	49.7	115.2
0	0	0						
length	50							

peak +8	R0	$1/\Theta_2$	inf	inflog	newly	newlylog	Reff	AttackRate
mean	60.1	163.	78 391.5	0.8	983.3	0.5	47.6	93.
median	59.9	156.5	734.5	0.8	192.4	0.4	47.3	90.6
Quantile .1	48.6	112.2	178.4	0.4	62.	0.2	40.4	77.6
Quantile .9	70.9	208.9	1766.1	1.2	669.5	0.8	54.8	113.3
0	0	0						
length	50							
